# An *in situ* visualization system using synchrotron white X-rays to investigate the solidification behaviors of metallic materials

**DOI:** 10.1107/S1600577525003716

**Published:** 2025-05-23

**Authors:** Hyeong Uk Mo, Min Woo Kim, Chang Hun Lee, Jina Kim, Hyun Wook Park, Jae-Hong Lim, Chang Hee Yim, Cheol Hee Nam, Jong Hyun Kim, Ho Jae Kwak

**Affiliations:** a Pohang Accelerator Laboratory (PAL) Pohang University of Science and Technology (POSTECH) Pohang37673 Republic of Korea; b Central Research Institute (CRI), Korea Hydro Nuclear Power (KHNP), Daejeon34101, Republic of Korea; c Department of Physics Kyungpook National University Daegu41566 Republic of Korea; d Graduate Institute of Ferrous and Eco Materials Technology Pohang University of Science and Technology (POSTECH) Pohang37673 Republic of Korea; e Technical Research Laboratory POSCO Gwangyang57812 Republic of Korea; f Department of Mechanical Engineering Pohang University of Science and Technology (POSTECH) Pohang37673 Republic of Korea; University of Essex, United Kingdom

**Keywords:** X-rays, metal and alloys, solidification, dendritic growth, *in situ*imaging, synchrotron X-rays

## Abstract

An advanced imaging platform has been developed to study the microstructural solidification behaviors of metals using synchrotron white X-rays. This system provides submicrometre effective pixel size and a frame rate of thousands per second, enabling high-resolution and high-speed imaging. The system functions independently, facilitating convenient alignment, magnification adjustments, and precise control of the region of interest. Additionally, we designed a specialized furnace for *in situ* characterization of microstructures during melting and solidification of metallic specimens at high temperature. This furnace meets stringent optical requirements and allows for finely adjusted specimen temperature gradients through the configuration of heating elements and individual current control. The furnace supports stable high-temperature experiments under vacuum, in an argon atmosphere, and at ambient pressure. Using this advanced imaging system, we investigated real-time *in situ* solidification phenomena of various metallic materials and other solidifying systems such as silicon. We performed image analysis to quantitatively assess microstructural changes, calculate dendritic spacing and determine liquid fractions.

## Abstract 2

This study introduces an advanced *in situ* visualization system using synchrotron white X-rays, enabling real-time imaging with micrometre resolution to investigate the solidification behaviors of metallic materials. These findings advance the understanding of microstructural evolution during melting and solidification, contributing to the development of materials with optimized properties.

## 1. Introduction

Metallic materials are widely used in automotive, construction, aerospace, marine, chemical and energy industries (Niinomi, 2002 ▸; Costa & Kenisarin, 2022 ▸; De Las Heras *et al.*, 2009 ▸; Czerwinski, 2021 ▸; Froes, 1994 ▸), due to their exceptional mechanical strength, superior thermal and electrical conductivities, robust corrosion resistance, and remarkable workability (Shi *et al.*, 2017 ▸; Xu *et al.*, 2015 ▸; Schweitzer, 2003 ▸; Gurrappa, 2003 ▸). These valuable characteristics are primarily determined by microstructures formed during solidification, including dendritic structures and grain morphology. Although the microstructure of solid metals can be further tailored through thermomechanical processes such as heat treatment, plastic deformation or recrystallization, in casting-based manufacturing the as-solidified microstructure plays a decisive role in defining the initial mechanical properties. Post-solidification treatments may enhance performance, but they often require additional processing steps, time and energy. Therefore, understanding and controlling microstructural evolution during solidification remains a critical objective in cast metal production (Quaresma *et al.*, 2000 ▸; Cáceres *et al.*, 2002 ▸; Zhang *et al.*, 2008 ▸; Osório & Garcia, 2002 ▸; Goulart *et al.*, 2006 ▸; Donelan, 2000 ▸; Filip *et al.*, 2003 ▸). A thorough understanding of microstructural characteristics, formation mechanisms, governing factors and control methods is essential to enhance desirable properties and optimize mechanical performance.

X-ray imaging enables non-destructive visualization of metallic materials’ internal structures and facilitates high-resolution, multi-scale microstructural analyses. Synchrotron X-rays, which are brighter and more coherent than laboratory X-rays, enable more efficient and precise evaluation. Notably, X-ray imaging using synchrotron white light does not require a dedicated monochromator, thereby ensuring higher photon flux. High photon energy greatly aids transmission imaging of thick or dense metallic materials. The increased flux enables clear imaging and rapid data collection, particularly during real-time *in situ* observations of dynamic changes.

Imaging studies of melting and solidification began in the 1960s. Early experimental efforts employed transparent compounds as analogs to investigate solidification behaviors such as the planar-to-dendritic transition and interface morphology under optical microscopy (Jackson & Hunt, 1965 ▸). These analog materials enabled indirect observation of phenomena otherwise obscured in metals due to opacity. Subsequently, *in situ* transmission electron microscopy (TEM) was used to directly observe melting and solidification phenomena in metallic systems, providing high-resolution insights into solid–liquid interface behavior, facet evolution and grain boundary dynamics (Glicksman & Vold, 1967 ▸). Laboratory X-rays revealed intricate high-resolution features of dendritic solidification structures (Forsten & Miekk, 1967 ▸). The development of third-generation synchrotron light sources and advanced optical instrumentation significantly accelerated real-time research concerning metallic materials. Notably, the first observation of dendritic solidification using synchrotron-based X-rays occurred in 1987 (Matsumiya *et al.*, 1987 ▸). This pioneering work began with observations of metal solidification behaviors in the Japanese KEK-EF facility, followed by detailed examinations of dendritic structure growth during solidification at various international facilities, including the LNLS in Brazil, the European ESRF-ID19 and the SRS-Daresbury Laboratory in the UK (Mathiesen *et al.*, 1999 ▸; Wang *et al.*, 2009 ▸; Grange *et al.*, 1994 ▸).

In particular, white-beam X-ray topography—made possible by synchrotron radiation—has emerged as a powerful tool for investigating crystal defects as well as conducting *in situ* studies of solidification and dendritic growth (Kwak *et al.*, 2023 ▸). This technique captures diffracted X-rays from multiple crystal planes simultaneously, forming characteristic Laue patterns that are sensitive to lattice orientation and phase transitions. This allows direct, real-time tracking of phase formation, crystallographic evolution and grain development during solidification.

Metallic material microstructures are profoundly influenced by thermal gradients, cooling rates and environmental conditions (Ares *et al.*, 2010 ▸; Boettinger *et al.*, 2000 ▸; Chen, 2002 ▸; Pocheau *et al.*, 2007 ▸; Gurevich *et al.*, 2010 ▸; Ghmadh *et al.*, 2014 ▸). Therefore, parallel to advances in real-time imaging, developing dedicated high-temperature furnaces that precisely control solidification and microstructural evolution during real-world processes are essential.

This paper describes the design and development of the white-beam-based X-ray imaging system at the 9D beamline of the Pohang Light Source-II (PLS-II), which facilitates *in situ*X-ray imaging. Additionally, we introduce a specialized heating and cooling furnace that aids real-time imaging of metallic material behaviors. Microstructural imaging during solidification of metals and alloys is described to demonstrate the effectiveness of both the imaging system and the furnace.

## 2. Instrumentation

### 2.1. White X-ray imaging equipment

The PLS-II is a third-generation synchrotron yielding X-rays with energy 3 GeV and current 400 mA (Lee *et al.*, 2022 ▸). The 9D beamline, using white light from a bending magnet, exhibits broad spectral characteristics and high flux, enabling rapid *in situ* imaging. The long distance between the light source and the sample ensures excellent spatial resolution (Pogany *et al.*, 1997 ▸). The approximate specifications of the photon source (full width at half-maximum values) are energy 4–50 keV, horizontal dimension (H) 60 µm, and vertical dimension (V) 30 µm. The beam size at the sample position (22 m from the source) can be controlled by several centimetres both horizontally and vertically. The most important beamline parameters are summarized in Table 1 ▸.

As X-rays penetrate a sample, they are converted into visible light by a scintillator, aiding detection. The visible light from the scintillator is reflected by a mirror through 90° from the X-ray beam path and then collected. Using optical lenses of various magnification ratios, the final images are captured by a highly sensitive camera. The spatial resolution and field of view (FOV) can be varied depending on the chosen combination of an optical objective lens, a scintillator and a camera.

In the scintillators, yttrium aluminium garnet (YAG) is used for high-resolution imaging based on its high light output (9000 photons MeV^−1^) and fast decay time (70 ns), whereas lutetium aluminium garnet is used for high-speed imaging because it emits brighter visible light than YAG at the same sample thickness due to its higher density. Visible light from the scintillators is combined with a microscopic objective lens and a complementary metal-oxide-semiconductor (CMOS) sensor to ensure submicrometre effective pixel size. Two types of CMOS cameras are installed: the pco.edge 5.5 (pixel size 6.5 µm and resolution 2560 × 2160 pixels) for high-resolution imaging and the pco.Dimax HS4 (pixel size 11 µm and resolution 2000 × 2000 pixels) for high-speed imaging. The FOV and effective pixel size can be adjusted by varying the chosen magnification lenses (×2, ×5, ×10 or ×20) to a maximum pixel size of 0.33 µm and an FOV of 0.83 mm × 0.70 mm. The specifications are shown in Table 2 ▸. The camera and lenses are mounted on independent stages to allow easy alignment. The sample stage exhibits both *Y*- and *Z*-axis substages to permit horizontal and vertical movements along the beam direction, with swivel and rotational substages on each axis to control the sample to vary the observational area.

### 2.2. High-temperature furnace for *in situ* experiments

We built a versatile furnace that meets stringent optical constraints and ensures precise atmospheric control during stable and reproducible heating and cooling. The performance of this furnace was verified using a previously developed prototype. Previous *in situ* studies using prototype furnace (Zargar *et al.*, 2023 ▸) and investigation at SPring-8 (Yasuda, Morishita *et al.*, 2020 ▸; Nishimura *et al.*, 2019 ▸; Yasuda, Suga *et al.*, 2020 ▸) confirmed significant deformations attributable to volumetric contractions caused by peritectic transitions during and at the end of carbon steel solidification. The furnace uses synchrotron X-rays to characterize dynamic behaviors of metals, particularly during melting and solidification, and examines metal microstructures. The newly developed furnace operates stably at high temperatures and allows precise control of temperature gradients and their directions.

The thermal stability of the furnace was verified indirectly through consistent dendritic growth behavior. Under unstable thermal conditions, dendritic growth typically exhibits irregular acceleration or deceleration. In contrast, the uniform growth rates observed throughout our experiments indicate a stable thermal environment during solidification. The outer coolant temperature was maintained at 15°C without observable fluctuation over extended operation. These combined observations confirm the furnace’s capability to maintain a reliable and thermally stable condition suitable for *in situ* solidification studies.

**Figure 1:**
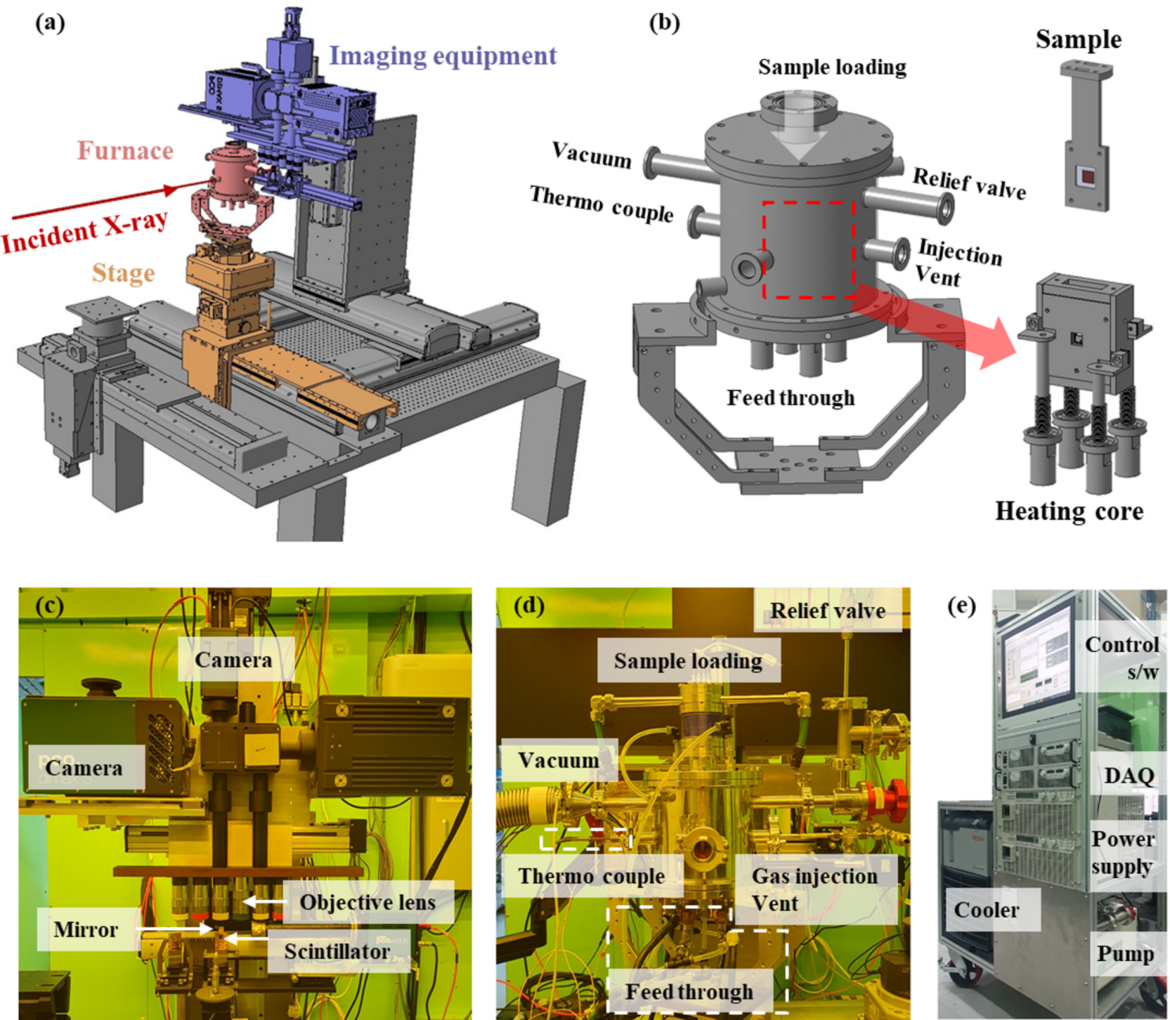
PLS-II 9D beamline, schematics and photographs of the visualization system for metallic materials using white X-rays. (*a*) Overview of the imaging and high-temperature furnace system, and (*b*) furnace concept. Photographs of the (*c*) imaging, (*d*) furnace and (*e*) associated equipment.

Schematics of the imaging and furnace system are shown in Fig. 1 ▸. A top insertion port accepts a sample. Visualization ports reveal incident, transmission and diffraction. Both sides feature ports for the vacuum pump, gas injection and exhaust, as well as a thermocouple port for temperature measurement. In addition, normal pressure or pressurized experiments are possible by connecting a relief valve. The furnace bottom exhibits four ports that fix the heating core and deliver current. The furnace is double chamber; cooling water circulates between the inner and outer sides. Precise control of heat applied to a sample is crucial to control microstructure melting, solidification and growth direction. The resistance element is heated using electric current, and this element then transfers heat to the sample in the form of radiation. The complex graphite heating element was designed using power density and resistance calculations to ensure efficient heating.

The *in situ* furnace utilizes graphite heaters placed on both the front and back sides of the sample. Each heater can be independently configured to operate on the left, right, top or bottom sides, allowing precise control of temperature gradients in multiple directions. While symmetric heater pairs are generally used for uniform heating, asymmetric combinations (*e.g.* front-left and back-right) enable tailored thermal fields across the sample. The configuration of the main heating elements (serving as radiation sources) can be flexibly adjusted to render the temperature gradient vertical, horizontal or uniform. The gradient magnitude is controlled by applying independent currents to each heating element, allowing fine-tuning of the thermal field. At high temperatures, this setup enabled precise control of temperature variations within ±1 K. The heating core uses porous carbon felt insulation and heat-refractory boron nitride to transfer and concentrate heat to the sample, ensuring that the chamber remains stably at room temperature even at high temperatures. In terms of cooling, the furnace supports a tunable cooling rate in the range 0.1–10 K s^−1^, measured from the onset of cooling in the high-temperature regime.

The peripheral equipment includes a vacuum pump, power supply, data acquisition system, cooler, control software and PC. To ensure integrated control, all components are installed in a single rack. To allow rapid achievement of melting temperatures and ensure temperature stability, PID software is utilized to acquire data and control outputs.

## 3. Results and discussion

Several experiments were conducted to observe the melting and solidification behaviors of metallic materials; we evaluated the performances of the optical imaging system and the heating furnace. Initially, melting of Inconel718 was studied to assess the furnace heating rate, temperature, cooling performance, stability and overall robustness. The Inconel718, Cu and Ni samples used in this study were all in the annealed condition, with purities of 99.90% for Cu and 99.00% for Ni. The Si sample was of electronic-grade purity (>99.999%). All samples were cut from foil and used without any additional surface treatment or modification.

To estimate the actual temperature experienced by the sample, a thermocouple was used to monitor the surrounding region near the sample. This thermocouple was calibrated through repeated melting experiments using various metallic materials with well known theoretical melting points.

**Figure 2:**
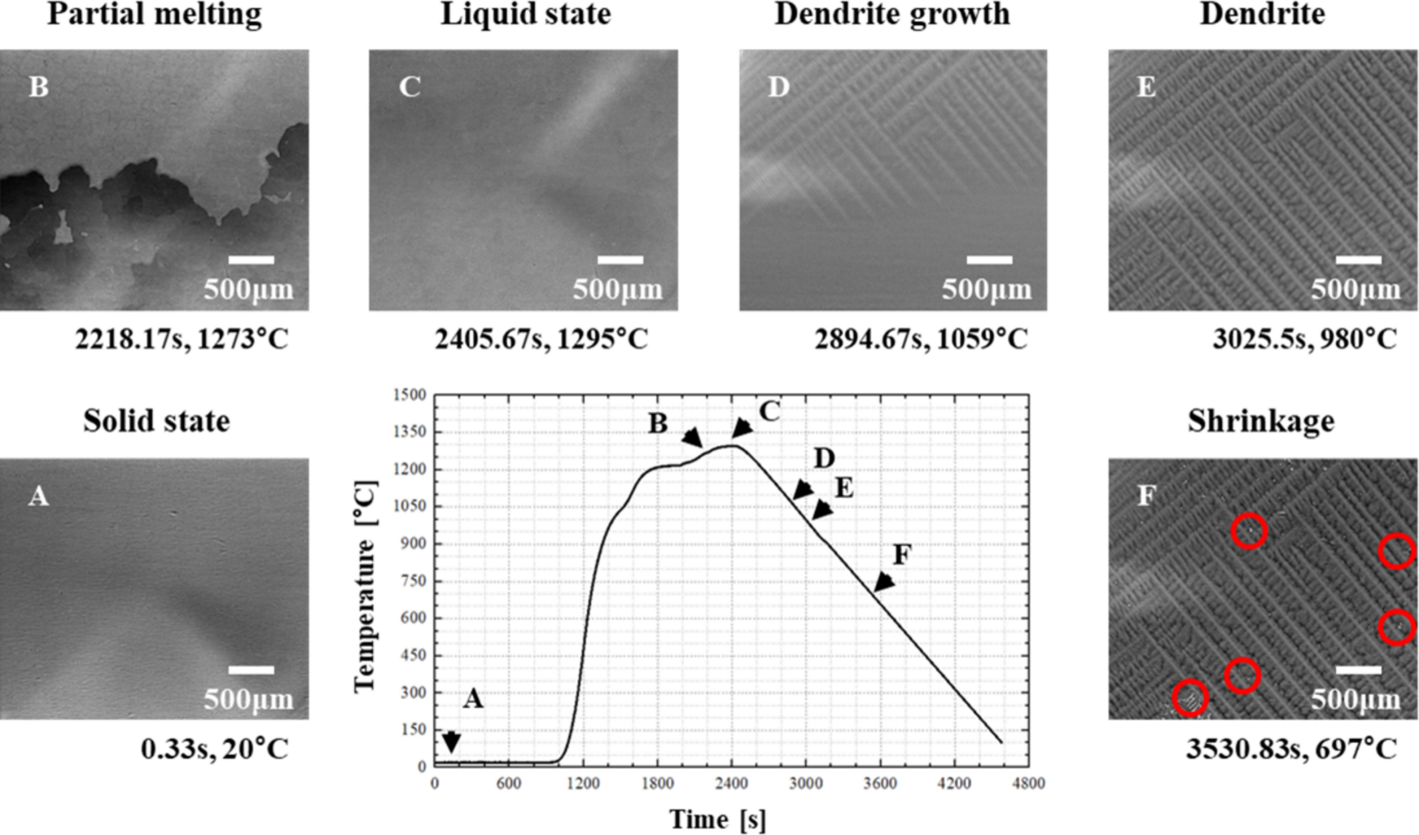
Phase changes of Inconel718 and dendritic and microstructural developments according to temperature.

Fig. 2 ▸ shows Inconel718 behaviors and microstructures at various temperatures. The entire melting/solidification process was captured using a high-resolution pco.edge 5.5 camera operating at 5× magnification, 20 ms exposure time and 5 frames s^−1^. The sample was heated to approximately 4 K s^−1^ with a current of approximately 5 A min^−1^ using a vertical temperature gradient heating core with an average resistance of 0.45 Ω. Melting, as estimated from the calibrated thermocouple near the sample, commenced approximately around 1250°C; after complete melting, the current decreased by 1 A min^−1^, resulting in cooling at approximately −0.4 K s^−1^.

In this experiment, a vertically oriented thermal gradient was established by configuring both the front and back heaters to have their primary heating elements at the bottom. As a result, melting initiated from the bottom where heat was concentrated, and solidification proceeded from the top down as the upper region, being farther from the heat source, cooled first. This behavior was clearly observed in time-resolved images and was consistent with the intended experimental design.

Except during outgassing, the chamber vacuum was maintained around 1.0 × 10^−5^ torr. Even during long-term repeat experiments, the cooling water temperature was 15°C; the furnace performed well at high operating temperatures, and the vacuum was never compromised.

**Figure 3:**
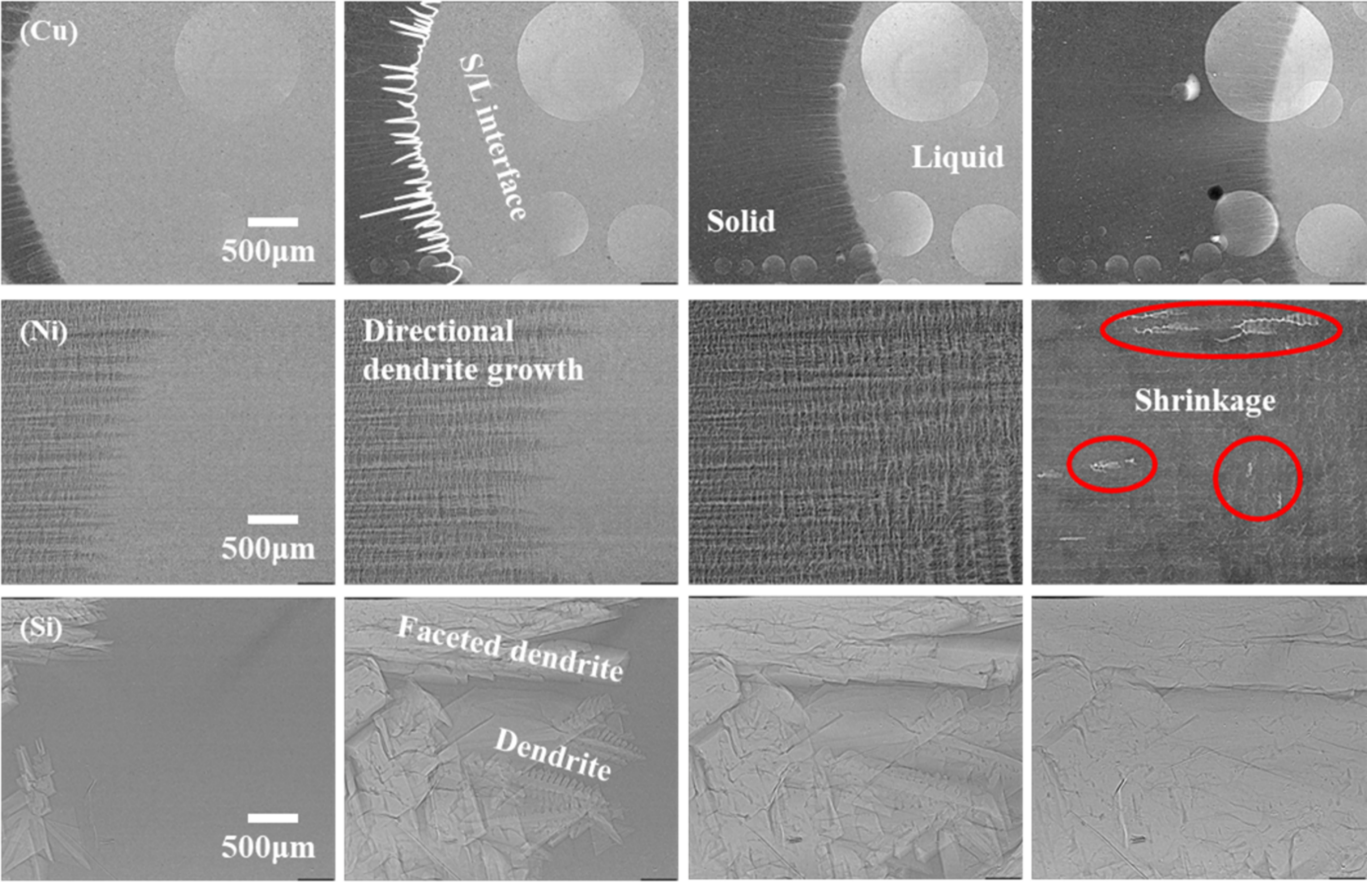
Dendrite and microstructural growth during the solidification processes of pure Cu, pure Ni and polycrystalline Si. (In the Cu sample, due to the presence of empty regions in the flat-field reference image, ring-like features appeared in the final frames. These are image artifacts and do not represent actual microstructural features.)

To verify the effect of temperature gradient directional changes afforded by the heater during imaging of various materials’ growth (under identical imaging conditions), microstructures were observed during the solidification of Cu, Ni and Si. The results are shown in Fig. 3 ▸. Using a horizontal temperature gradient heating core, the temperature increased at 4 K s^−1^ and, after confirming complete melting, decreased at approximately 0.5 K s^−1^. During directional solidification of Cu, Ni and Si, most dendritic structures ran parallel to the heat flow direction, oriented toward the opposite (right) side, confirming the horizontal temperature gradient.

All projection images were corrected using flat-field and dark-field normalization to reduce detector-related noise and enhance image contrast. To highlight the interface between the solid and liquid phases, the flat-field reference image was acquired during the fully molten (full-liquid) state of the sample.

In addition to the solidification front evolution, shrinkage-induced porosities—an inherent feature of volume contraction during solidification—were also clearly captured, as highlighted by the red circles in Figs. 2 ▸ and 3 ▸, demonstrating the system’s capability to visualize critical microstructural phenomena in real time.

These specific observations suggest that our system is capable of capturing a wide range of solidification behaviors and microstructural phenomena, such as the columnar-to-equiaxed transition (Reinhart *et al.*, 2005 ▸; Gandin, 2000 ▸; Dong & Lee, 2005 ▸), dendrite fragmentation (Mathiesen *et al.*, 2006 ▸; Chang *et al.*, 2012 ▸), dendrite interaction of branches (Bogno *et al.*, 2013 ▸) and secondary dendrite branching, as documented in previous literature.

**Figure 4:**
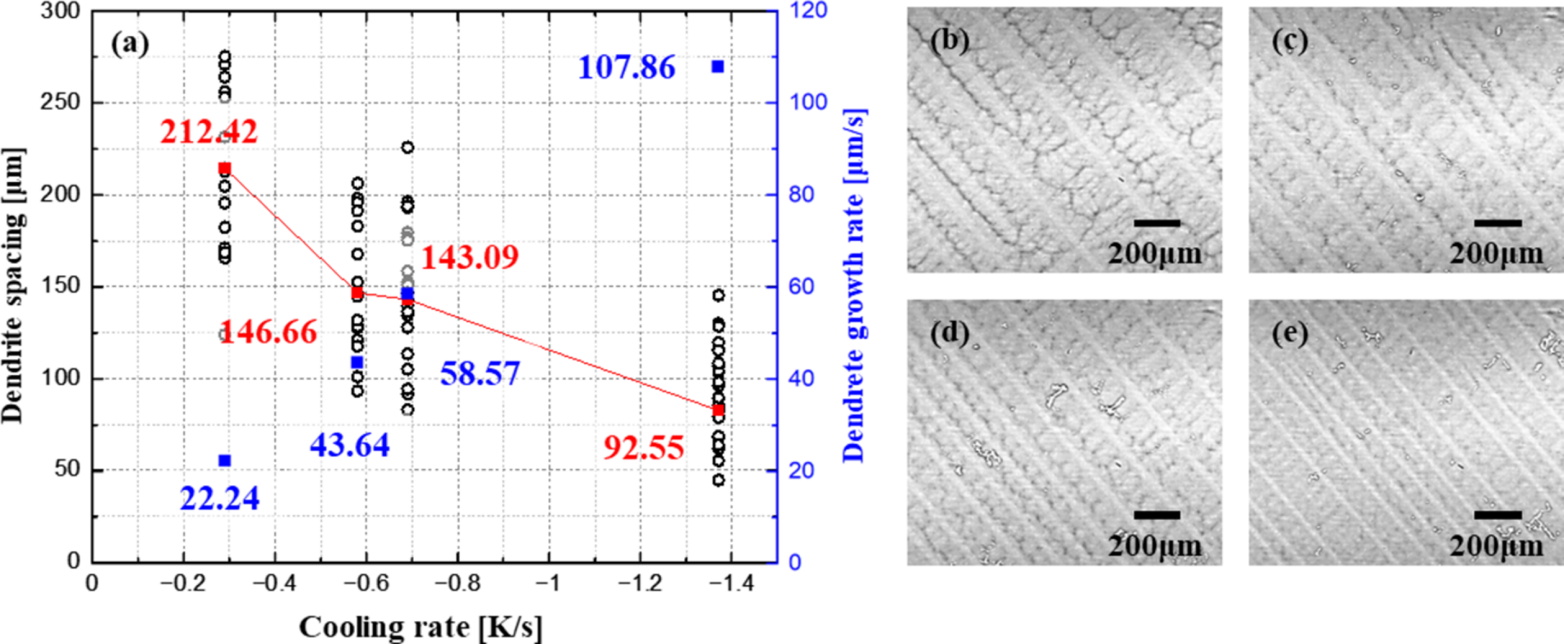
(*a*) Dendrite spacing (in red) and dendrite growth rate (in blue) as a function of cooling rate, as determined from synchrotron imaging: (*b*) −0.29 K s^−1^, (*c*) −0.58 K s^−1^, (*d*) −0.69 K s^−1^ and (*e*) −1.37 K s^−1^.

Dendrite growth behavior was quantitatively analyzed under various controlled cooling rates using time-resolved synchrotron imaging. The current was decreased by 0.5, 1, 2 and 5 A min^−1^, corresponding to cooling rates of −0.29, −0.58, −0.69 and −1.37 K s^−1^, respectively. Representative dendritic structures of solidified Inconel718 at each cooling rate are shown in Fig. 4 ▸. At each cooling condition, both dendrite growth rate and primary dendritic spacings were measured. The growth rate increased consistently with cooling rate, from 22.24 µm s^−1^ at −0.29 K s^−1^ to 43.64 µm s^−1^ at −0.58 K s^−1^, 58.57 µm s^−1^ at −0.69 K s^−1^ and 107.86 µm s^−1^ at −1.37 K s^−1^. Correspondingly, dendritic spacings decreased with increasing cooling rate: at the lowest cooling rate, spacing ranged from 124 to 275 µm (average 212 µm), while higher cooling rates yielded average spacings of 146.66, 143.09 and 92.55 µm, respectively. These observations demonstrate a clear correlation between cooling rate and both growth kinetics and microstructural scale, highlighting the system’s capability to capture and quantify dynamic solidification processes with high spatial and temporal resolution. Moreover, the dendrite growth rate increased in near-proportional response to the applied cooling rate, and this consistent trend, observed across multiple trials, confirms the system’s high degree of experimental reproducibility under controlled thermal conditions.

Even minor variations in reaching the precise melting temperature or instances of overheating followed by rapid cooling can suppress dendritic growth, alter growth velocities or change morphologies. However, in our experiments, dendritic structures consistently developed with predictable kinetics and forms, demonstrating both thermal stability and reproducibility of the system.

Additionally, images of growing dendrites were analyzed to obtain data regarding the liquid fraction. It can be used to distinguish and analyze the liquid and solid states of metals or alloys apparent on X-ray transmission images. The Beer–Lambert equation was rewritten as an equation yielding the densities of solids and liquids evident in X-ray images, then used to calculate the liquid fraction,
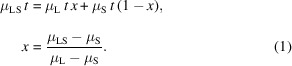
Here, *x* (the liquid fraction) represents the proportion of the sample in the liquid state. μ_LS_*t* is the product of the linear attenuation coefficient and thickness of the mixed solid and liquid state. μ_S_*t* is the product of the attenuation coefficient and thickness of the solid state, whereas μ_L_*t* is the corresponding parameter for the liquid state. The formula utilizes the attenuation coefficient and thickness in the mixed state to calculate the liquid fraction *x* by comparing the above-mentioned values with those of the solid and liquid states. It is possible to qualitatively derive the proportions of solid and liquid in a sample, greatly aiding the understanding of roles played by various states during metal melting and solidification.

**Figure 5:**
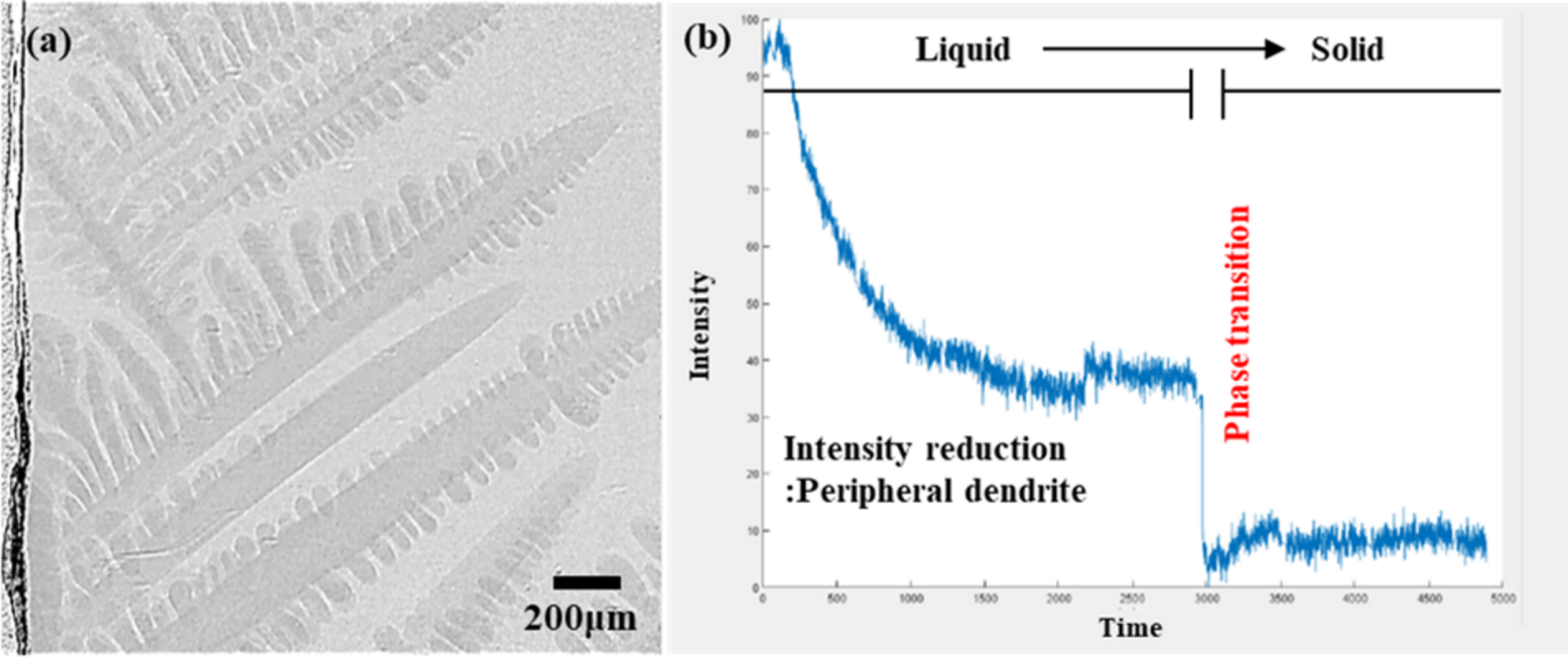
(*a*) Dendrite images of the solid–liquid fraction during solidification, and (*b*) corresponding changes in liquid fraction over time.

Fig. 5 ▸ shows the results obtained by the experimental and analytical methods. We used a pco.edge 5.5 camera operating at 20× magnification and analyzed post-processed *in situ* images to determine liquid fractions during the phase transition from liquid to solid. The liquid-to-solid transition greatly affected brightness; the liquid fraction rapidly changed as dendrites grew over time.

We developed a versatile imaging and furnace system using synchrotron white X-rays for *in situ* characterization of metal microstructures during melting and solidification. The PLS-II 9D beamline imaging system employs high-intensity white X-rays that capture dynamic metal microstructural changes at micrometre-resolution using high frame rates. The furnace ensures a meticulously controlled and reproducible experimental environment; precise thermal regulation is provided by the current delivered to the graphite heating elements. The system accommodates vertical, horizontal, uniform and extreme temperature gradients, operating stably over time at high temperatures.

## 4. Conclusion

In this study, we developed and demonstrated an advanced *in situ*X-ray imaging system coupled with a precisely controllable high-temperature furnace for real-time observation of microstructural evolution during metal melting and solidification. The system utilizes high-flux synchrotron white X-rays to capture dendritic growth, phase transitions and porosity formation with high spatial and temporal resolution. The furnace allows flexible thermal configurations and stable operation, enabling controlled experiments across a wide range of temperature gradients and cooling rates.

Through systematic experiments, we confirmed clear correlations between cooling rates and dendritic growth kinetics, spacing and liquid fraction evolution. Moreover, the system successfully visualized key solidification phenomena such as directional growth, shrinkage porosity and microstructural transitions, thereby validating its capability for dynamic, quantitative analyses. Notably, the system consistently reproduced fine-scale dendritic growth under controlled thermal conditions, confirming its high degree of experimental reproducibility.

Overall, the proposed platform presents a robust and versatile tool for fundamental and applied research in solidification science. It provides valuable insights into the thermal and microstructural behaviors of metallic materials and holds strong potential for future applications in alloy design, casting process optimization and advanced materials development.

## Figures and Tables

**Table 1:** Beamline parameters

Type	Bending magnet
Electron energy	3 GeV
Stored electric current	400 mA
X-ray wavelength	4–50 keV
X-ray photon flux at sample	10^12^ photons s^−1^ (0.1% bandwidth)^−1^
Beam size at source	60 µm (H) × 30 µm (V)
Beam size at sample	8 mm (H) × 5 mm (V)

**Table 2:** Theoretical image resolutions

Edge 5.5	Dimax HS4
16.64 mm × 14.04 mm	11 mm × 11 mm
6.5 µm × 6.5 µm	22.00 µm × 22.00 µm
2560 × 2160 pixels	2000 × 2000 pixels
100 frames s^−1^ (maximum)	2277 frames s^−1^ (maximum)
30000:1 (maximum)	2000:1 (65 dB, CDI)
